# Integrating GWAS and Transcriptomics to Identify the Molecular Underpinnings of Thermal Stress Responses in *Drosophila melanogaster*

**DOI:** 10.3389/fgene.2020.00658

**Published:** 2020-06-23

**Authors:** Melise C. Lecheta, David N. Awde, Thomas S. O’Leary, Laura N. Unfried, Nicholas A. Jacobs, Miles H. Whitlock, Eleanor McCabe, Beck Powers, Katie Bora, James S. Waters, Heather J. Axen, Seth Frietze, Brent L. Lockwood, Nicholas M. Teets, Sara H. Cahan

**Affiliations:** ^1^Department of Entomology, University of Kentucky, Lexington, KY, United States; ^2^Department of Biology, University of Vermont, Burlington, VT, United States; ^3^Department of Biology, Providence College, Providence, RI, United States; ^4^Department of Biology and Biomedical Sciences, Salve Regina College, Providence, RI, United States; ^5^Department of Biomedical and Health Sciences, University of Vermont, Burlington, VT, United States

**Keywords:** thermal limit, CT_min_, CT_max_, heat shock, cold shock, genomics, transcriptomics

## Abstract

Thermal tolerance of an organism depends on both the ability to dynamically adjust to a thermal stress and preparatory developmental processes that enhance thermal resistance. However, the extent to which standing genetic variation in thermal tolerance alleles influence dynamic stress responses vs. preparatory processes is unknown. Here, using the model species *Drosophila melanogaster*, we used a combination of Genome Wide Association mapping (GWAS) and transcriptomic profiling to characterize whether genes associated with thermal tolerance are primarily involved in dynamic stress responses or preparatory processes that influence physiological condition at the time of thermal stress. To test our hypotheses, we measured the critical thermal minimum (CT_min_) and critical thermal maximum (CT_max_) of 100 lines of the *Drosophila* Genetic Reference Panel (DGRP) and used GWAS to identify loci that explain variation in thermal limits. We observed greater variation in lower thermal limits, with CT_min_ ranging from 1.81 to 8.60°C, while CT_max_ ranged from 38.74 to 40.64°C. We identified 151 and 99 distinct genes associated with CT_min_ and CT_max_, respectively, and there was strong support that these genes are involved in both dynamic responses to thermal stress and preparatory processes that increase thermal resistance. Many of the genes identified by GWAS were involved in the direct transcriptional response to thermal stress (72/151 for cold; 59/99 for heat), and overall GWAS candidates were more likely to be differentially expressed than other genes. Further, several GWAS candidates were regulatory genes that may participate in the regulation of stress responses, and gene ontologies related to development and morphogenesis were enriched, suggesting many of these genes influence thermal tolerance through effects on development and physiological status. Overall, our results suggest that thermal tolerance alleles can influence both dynamic plastic responses to thermal stress and preparatory processes that improve thermal resistance. These results also have utility for directly comparing GWAS and transcriptomic approaches for identifying candidate genes associated with thermal tolerance.

## Introduction

Temperature directly affects performance, survival, fitness, and consequently, the geographic distribution of organisms (Angilletta, 2009; Dowd et al., 2015). Ectotherms are particularly vulnerable to changes in temperature, and these organisms have evolved a suite of adaptations to cope with thermal variability. An ectotherm’s thermal tolerance is determined by both fixed genetic factors and plastic changes in behavior, morphology, physiology, and gene expression. Genetic variation in thermal tolerance is well-documented (e.g., Sørensen et al., 2001; McMillan et al., 2005; Rako et al., 2007) and can occur through changes in basal stress tolerance and/or changes in the ability to quickly respond to thermal challenges (Ayrinhac et al., 2004). These heritable differences within populations permit evolutionary shifts in thermal response as selection acts (Hoffmann et al., 2003), and adaptive differences in thermal tolerance across latitudinal gradients and thermal environments are common (Hoffmann et al., 2002; Fallis et al., 2014). Specifically, populations from higher latitudes often are more tolerant of low temperatures than populations from lower latitudes, and the same pattern is also seen for heat stress, where populations that extend to lower latitudes often have improved survival at high temperatures (e.g., Calosi et al., 2010; but see Castañeda et al., 2015). Thus, thermal tolerance is a trait that is both highly plastic and highly adaptable, and understanding the genetic basis of thermal tolerance is critical for predicting future responses to environmental change.

Genome Wide Association Studies (GWAS) and other quantitative genetic approaches have characterized the genetic architecture of thermal tolerance and identified genes that regulate temperature-dependent traits (e.g., Morgan and Mackay, 2006; Rako et al., 2007; Svetec et al., 2011; Rohde et al., 2016). A series of recent studies with the *Drosophila* Genetic Reference Panel (DGRP; Mackay et al., 2012) have identified a number of candidate loci associated with thermal tolerance. Rolandi et al. (2018) found 12 SNPs associated with variation in critical thermal maximum (CT_max_), and most of these SNPs were located within intronic regions, suggesting that variation in the heat stress response could be mediated by regulatory changes in gene expression or splicing. For cold, distinct but related traits often have non-overlapping genetic architectures, suggesting these traits have the capacity to evolve independently. For example, two plastic responses to cold, rapid cold hardening and developmental cold acclimation, have non-overlapping SNPs associated with them, although the genes associated with these traits share some functional similarities (Gerken et al., 2015). Similarly, Teets and Hahn (2018) found minimal overlap in genes associated with cold shock response and chill coma recovery, and Freda et al. (2017) found no overlap in genes associated with adult and larval cold hardiness. The candidate genes identified in Teets and Hahn (2018) were functionally tested with RNAi, and knockdown of most genes affected cold tolerance, indicating that GWAS is a robust method for identifying genes with functional roles in thermal tolerance. Taken together, the various GWAS studies of thermal traits indicate that the thermal stress response is a highly polygenic trait, but additional studies linking these polymorphisms to their functional consequences are needed to clarify their role in thermal tolerance.

One way the genetic makeup of an organism influences thermal tolerance is by modifying gene expression changes in response to temperature change (Stucki et al., 2017). Transcriptional responses to thermal variability have been described at various levels, including whole transcriptomic studies of specific stress treatments (e.g., Qin et al., 2005; Sørensen et al., 2005, 2016; Teets et al., 2012), targeted experiments for specific candidate genes (e.g., *Frost* in Goto, 2001; Sinclair et al., 2007; Zhu et al., 2017), and comparisons of transcriptomic responses to thermal stressors both among (e.g., in damselflies; Lancaster et al., 2016) and within populations (e.g., Telonis-Scott et al., 2009). A consistent theme from these studies is that changing temperatures can cause substantial changes in gene expression. For example, in *D. melanogaster*, acclimation that enhanced the cold response led to nearly one third of the transcriptome being differentially regulated (with around 60% of these genes being downregulated; MacMillan et al., 2016). This cold acclimation included upregulation of genes already known to have an association with stress and temperature responses, such as *Frost* and many genes encoding for heat shock proteins. Similar sets of genes are also upregulated following brief cold shock in *D. melanogaster* and the flesh fly *Sarcophaga bullata* (Qin et al., 2005; Teets et al., 2012), indicating that anticipatory acclimation responses share some mechanisms with dynamic responses that occur during and after stress. For heat stress, most genes that are differentially expressed following short-term heat hardening (Sørensen et al., 2005) and heat shock (Telonis-Scott et al., 2013) in *D. melanogaster* are downregulated, with the exception of heat shock proteins, which are generally upregulated. However, despite the large body of literature on transcriptional responses to thermal stress, additional work is needed to clarify the functional consequences of these transcriptomic changes and determine how segregating variation in thermal tolerance relates to these transcriptional mechanisms.

Thermal tolerance is a combination of dynamic plastic changes that occur during and after a stress event (i.e., processes that actively counter, repair or minimize the consequences of damage) and preparative processes that enhance stress resistance (i.e., processes that prevent damage; Roy and Kirchner, 2000; Wos and Willi, 2015). Plastic processes that occur during and after thermal stress largely involve production of stress proteins (e.g., heat shock proteins), often at the expense of other biological processes (Feder and Hofmann, 1999). Preparative processes that enhance thermal resistance include production of protective osmolytes (e.g., cryoprotectants; Yancey, 2005; Storey and Storey, 2012), changes in membrane composition and cell structure that permit membrane function at extreme temperature (Sinensky, 1974; Koštál, 2010), and anticipatory production of stress proteins during dormancy and/or thermal acclimation (Manjunatha et al., 2010; Colinet et al., 2013; MacMillan et al., 2016). Thus, an allelic variant may contribute to basal tolerance to extreme temperature by altering either of these two components: enhancing the plastic ability to adjust to stress by participating in or regulating the dynamic temperature response, or by better preparing the organism for that stress. However, for genes associated with variation in thermal tolerance, whether these genes primarily affect dynamic plastic processes or preparative processes is unclear.

Here, we used a combination of GWAS and RNA-seq to address the extent to which genes associated with thermal tolerance variation are involved in the dynamic stress response and preparative responses. We measured critical thermal minimum (CT_min_) and CT_max_ (Schou et al., 2017) in 100 lines from the DGRP, and the resulting phenotypic data were used in conjunction with genome-wide polymorphism data to identify genes associated with variation in thermal limits. These candidate genes were then compared to differentially expressed genes identified via transcriptomic assays of a single genotype exposed to heat and cold shock treatments to identify their roles in the stress response. Three non-mutually exclusive hypotheses were considered. To identify candidates involved in dynamic stress responses, we tested the following two specific hypotheses: (H1) Genes associated with thermal tolerance are part of the dynamic response, and are directly up- or downregulated during thermal stress; (H2) Genes associated with thermal tolerance are transcription factors and regulatory genes that regulate the dynamic transcriptional response to thermal stress. In support of H1, we predict that GWAS candidates will be more likely to be up- or downregulated in response to thermal stress, and these candidates will include genes directly activated during the stress response (e.g., heat shock proteins) as well as genes downregulated because they are incompatible with stressful temperatures (e.g., certain metabolic processes and reproduction). To identify genes involved in preparative processes that enhance thermal stress resistance, we tested the following hypothesis: (H3) Genes associated with thermal tolerance are involved in preparatory developmental and physiological processes that influence the condition of the organism at the time of thermal stress. Here we predict that specific GWAS candidates will be involved in the stress resistance processes discussed above (e.g., cell membrane remodeling, osmolyte production, etc.), but these candidates will not necessarily be part of the dynamic stress response. Our results indicate that genes associated with the thermal response have diverse functional roles that contribute to thermal tolerance in all three of these ways. There is considerable overlap between the genes associated with quantitative variation in thermal tolerance and those that are differentially expressed in response to thermal stress, and our GWAS analysis indicated an abundance of genes involved in developmental processes and cell morphogenesis that may have a role in enhancing stress resistance. Testing these hypotheses will advance our understanding of the functional consequences of genes polymorphisms associated with thermal tolerance. Furthermore, these results also have utility for directly comparing two commonly used methods for identifying and characterizing candidate genes associated with thermal tolerance.

## Materials and Methods

### Insect Rearing

The *Drosophila* Genetic Reference Panel (DGRP) was established from a natural population in Raleigh, North Carolina, and isofemale lines were isogenized with 20 generations of full-sib mating (Mackay et al., 2012). DGRP lines were obtained from the Bloomington Drosophila Stock Center, maintained at 25°C on a 12:12 light–dark cycle, and fed a standard cornmeal/soy flour diet consisting of 0.58% agar, 1.73% yeast, 7.31% cornmeal, 1.00% soy flour, 0.13% Tegosept (w/v), 7.69% light corn syrup, and 0.48% propionic acid (v/v) in H_2_O. To generate flies for CT_min_ and CT_max_ assays, 15 females and 10 males were added to vials containing food and dry active yeast and were allowed to mate and lay eggs for 4 days. Restricting the number of adults in each vial and limiting the time to lay eggs prevented vials from becoming overcrowded, as extremely high larval densities can impact thermal tolerance (Sørensen and Loeschcke, 2001). Ten days after removing the parental adults, adults of the resulting progeny were removed and held for 24 h to ensure that all flies had an opportunity to mate. After 24 h, males and females were sorted and placed into separate vials in groups of 20. Flies were held in the vials for 3–4 days prior to measuring CT_min_. For CT_max_, flies were held for 2–3 days, and 24 h prior to the experiment, flies were lightly anesthetized with CO_2_ and individually transferred to small screw-top vials with food. All flies were between 4 and 9 days old at the time of the experiment.

Seven-day-old *D. melanogaster* Canton-S female flies were used to characterize gene expression responses after a cold or heat shock. Only females were used to minimize confounding variation due to sex differences in gene expression and to include gene expression responses associated with protection of egg production, which is strongly related to fitness and may be expected to be under selection in nature. We selected the Canton-S background for these experiments to (1) address the extent to which GWAS candidates predict gene expression in a standard, well-characterized genetic background, to increase the generalizability of our results, and (2) provide candidate genes for future functional experiments, as most mutant and transgenic strains are in the Canton-S background. To generate flies of known age for RNA-seq assays, all adults were removed from mixed-sex stock vials that had been maintained at approximately 50 flies per vial and all newly eclosed adults were sampled daily. Same-day cohorts were maintained in mixed-sex vials at a density of ∼30 flies/vial on a nutrient-rich medium, consisting of 0.88% agar, 8.33% yeast, 10% cornmeal, 0.33% Tegosept (w/v), 4.66% molasses, and 0.66% propionic acid (v/v) in dH_2_O (Buchanan et al., 2018), at 25°C on a 12:12 light–dark cycle. Cohorts were transferred to fresh food vials after 4 days.

### Phenotypic Assays

To measure CT_min_ and CT_max_, we used a dynamic ramping approach in which flies were gradually cooled or heated until motor function was lost. To assess CT_min_, we used a vertical jacketed column (modified from Huey et al., 1992) connected to a temperature-controlled fluid bath, and the temperature was monitored inside the column with a type T thermocouple (Supplementary Figure 1A). For detailed assembly instructions for the jacketed column, see Awde et al. (2020). For each line, ∼20 males and ∼20 females were combined in the column and submitted to the following thermal program: 25°C for 5 min, 25°C to 10°C at 0.5°C min^–1^, 10°C for 2 min, then 10°C to −10°C at 0.25°C min^–1^. The ramping rates are in line with other studies of CT_min_ and were designed to maximize throughput and prevent cold hardening during the procedure (e.g., Sinclair et al., 2015). At 10°C we began collecting flies as they reached their CT_min_ and fell through the column into collection vials containing 70% ethanol. New vials were placed under the column at 0.25°C intervals as the temperature decreased. Flies were typically at the top or on the walls of the column at the beginning of a trial (since they are negatively geotropic), and any flies that remained at the bottom of the column were discarded once the temperature reached 10°C. Flies from each vial were then sexed and counted, and the CT_min_ was recorded as the maximum temperature for a given interval (e.g., flies collected between 10°C and 9.75°C had a CT_min_ of 10°C). CT_min_ for each line was estimated by averaging the CT_min_ of all flies tested across two independent cohorts. Due to variation in line productivity, escaping flies, and discarded flies, the total number of flies measured per line ranged from 15 to 44 for males (median = 28 and mode = 28) and from 9 to 38 for females (median = 26 and mode = 26).

CT_max_ was assessed using the same apparatus as CT_min_, except the jacketed column was arranged horizontally and flies were contained individually in 2 ml screw-top vials to prevent them from voluntarily walking out of the column as temperature increased (Supplementary Figure 1B). For each line, ∼18 males and ∼18 females were individually placed in vials attached to a wooden dowel (Supplementary Figure 1B). The wooden dowel with the vials was placed inside the column and submitted to the following ramping program: 25°C for 5 min, 25°C to 35°C at 0.5°C min^–1^, then 35°C to 45°C at 0.25°C min^–1^. Flies were checked for movement after the temperature reached 35°C by flicking the wooden dowel every 0.2°C. The CT_max_ of flies was recorded when flies were motionless and no longer responded to stimulus. As with CT_min_, CT_max_ for each line was estimated by averaging the CT_max_ of all flies tested across two independent cohorts. Due to variation in line productivity and escaping flies, the total number of flies per line ranged from 23 to 53 for males (median = 33 and mode = 33) and from 24 to 51 for females (median = 33 and mode = 32).

We also tested the extent to which CT_min_ and CT_max_ were correlated with other life-history parameters and other measures of thermal tolerance using previously collected phenotype data for the DGRP. We obtained data for lifespan and fecundity from Durham et al. (2014), *Wolbachia* infection status and chill coma recovery time from Mackay et al. (2012), rapid cold hardening and chronic and acute survival from cold from Gerken et al. (2015), CT_min_ from Ørsted et al. (2018), cumulative cold tolerance from Teets and Hahn (2018), heat knockdown from Rohde et al. (2016), CT_max_ from Rolandi et al. (2018), and cold and heat hardness from Freda et al. (2019). We used Pearson correlations (*cor.test*) to test for linear correlation between these measures in R (version 3.6.1; R Core Team, 2019).

### Heritability and Genome Wide Association Study (GWAS)

Broad sense heritability (*H*^2^), defined as the proportion of the total phenotypic variation that is due to all genetic factors, was estimated as *H*^2^ = σ^2^_L_/(σ^2^_L_ + σ^2^*_ε_*), where σ^2^_L_ is among-line and σ^2^*_ε_* is within-line variance components (Mackay and Huang, 2018). Variance components were estimated using a linear mixed model and treating line as a random effect, with the *lme4* package (Bates et al., 2015) in R.

Genome wide associative mapping was used to identify genetic polymorphisms associated with CT_min_ and CT_max_ using the GWAS platform available on the DGRP website^1^ (Mackay et al., 2012). This analysis associates the phenotypic variation of DGRP lines with single nucleotide polymorphisms (SNPs), insertions, deletions, and multiple nucleotide polymorphisms (MNPs). One of the lines tested (208) was removed from the GWAS analysis by the DGRP server, thus the GWAS analysis included 99 lines. Variants with *p*-value < 1E-4 (using the average mixed *p*-value of the two sexes) were considered significant and were annotated to genes using FlyBase annotation v5.57. The average mixed *p*-value of the two sexes for GWAS analysis was chosen because both CT_min_ and CT_max_ were significantly correlated across sexes (see the section “Results”). To identify transcriptional regulators of thermal stress in support of H2 (see the section “Introduction”), we compared GWAS candidates to annotated transcription factors in FlyBase annotation v5.57.

As an alternative to GWAS on individual variants, we also conducted gene-based GWAS to test the aggregated effect of a set of SNPs (e.g., SNPs within a gene) on CT_min_ and CT_max_ phenotypes. Gene-based *p*-values were calculated by contrasting the observed T value to an empirical distribution generated from resampling under the null hypothesis with permutations using PLINK (version 1.9; Purcell et al., 2007; Liu et al., 2010). We controlled for confounding genetic relatedness between the DGRP lines used in this study, and used the Tracy-Windom test in the AssocTests package (Wang et al., 2015) to evaluate eigenvalues from 20 principal components (PCs) of genotype. We retained the first eight PCs as covariates in the PLINK model as described above (Patterson et al., 2006). As inversions and *Wolbachia* infection status can also influence the phenotypes of the DGRP lines, we used the adjusted phenotypes for these factors outputted from the DGRP2 website. Variants with MAF ≥ 5% and a genotype rate of 20% were used as well as the FlyBase v5.57 gene annotations. A total of 1,939,313 variants were tested over 8,954 and 8,270 genes with at least one significant variant for CT_min_ and CT_max_, respectively. Genome-wide significance was determined by controlling for FDR using the q value method (Storey and Tibshirani, 2003).

### RNA-Sequencing and Differential Gene Expression

To characterize gene expression responses to thermal shock, three replicates of three females each were exposed to cold or heat shock conditions by placing flies in sealed 15 × 150 mm glass test tubes and submerging in a circulating water bath programmed to cool or heat at a rate of 0.25°C min^–1^ until the temperature reached 4°C or 37°C, respectively. Flies were held at the final temperature for 5 min and then collected into pre-filled bead homogenization tubes (Benchmark Scientific) under CO_2_ anesthesia, immediately snap-frozen in liquid nitrogen and held at −80°C. Control flies were similarly handled but remained at 25°C until collection and flash-freezing. Whole flies were homogenized using a Bullet Blender Bead Homogenizer (Next Advance) in 300 μL TRIzol Reagent (Life Technologies) followed by purification with a Direct-zol RNA MicroPrep Kit (Zymo Research #R2060) according to the manufacturer’s instructions. DNA was removed using DNAse I on the column followed by two washes with RNA Wash Buffer. Total RNA was eluted with 15 μL DNAse/RNAse-free water, quantified using a Qubit 2.0 Fluorometer (Thermo Fisher), and RNA quality was assessed using an Agilent 2100 Bioanalyzer (Agilent Technologies). RNA-seq libraries were generated from 1 ug of rRNA-depleted total RNA using the NuQuant Universal RNA-Seq Library Preparation Kit (Nugen #M01506) according to the manufacturer’s protocol with 12 cycles of PCR. A total of nine libraries were pooled and sequenced on a single lane of an Illumina HiSeq1500 single-read flow cell. The sequence quality of the resulting raw Illumina reads was assessed using FastQC (version 0.11.4) and reads were aligned to the *D. melanogaster* reference genome (Release 6) using STAR aligner (Dobin et al., 2013). Genes were quantified using featureCounts (part of the Rsubread package, version 2.0.0, Liao et al., 2019) against the DM6 build. Differential expression was performed using the DESeq2 package (version 1.24.0; Love et al., 2014) in R. All data have been deposited into the NCBI SRA database with accession Bioproject PRJNA612361.

### Pathway Enrichment Analysis

Overrepresentation analysis (ORA) of significantly differentially expressed genes (Benjamini–Hochberg corrected *p*-value < 0.01, fold-change > 2) and the GWAS genes (*p*-value < 1E-4) was performed using WebGestalt (Wang et al., 2017; minimum five genes per category, maximum 2,000 genes) and a false discovery rate cut-off of 0.05. The results of the overrepresentation analysis were the primary means by which we identified GWAS candidates in support of H3 (see the section “Introduction”).

### Integration of Differential Gene Expression With GWAS

Genes associated with SNPs identified using GWAS were matched with corresponding genes in the expression data set. For cases where multiple genes were associated with a single SNP, each gene was included. The distribution of log-fold changes of the expression of all genes in the heat shock and cold shock versus control was compared to the fold-change distribution of the genes significantly associated with the corresponding thermal performance limit with Kolmogorov–Smirnov tests implemented in R. These analyses were the primary means by which we identified inducible genes in support of H1 (see the section “Introduction”).

## Results

### Genetic Variation in Thermal Tolerance

**FIGURE 1:**
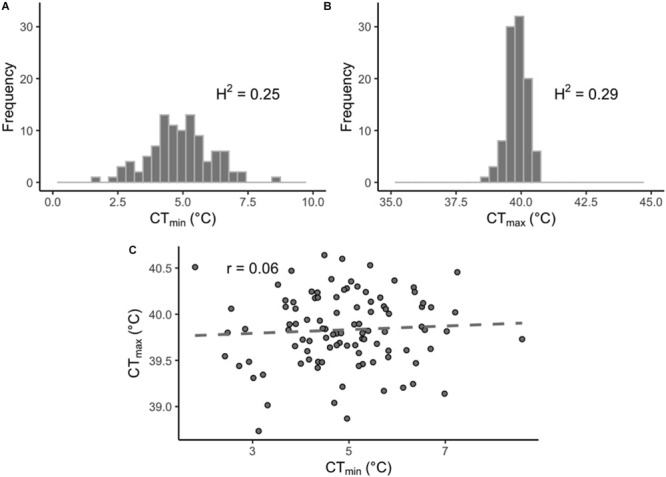
Distribution of thermal limits in the DGRP. Histograms of mean phenotypes across 100 DGRP lines for **(A)** CT_min_ and **(B)** CT_max_. **(C)** Correlation between line-means of CT_min_ and CT_max_.

In this study we measured thermal limits (CT_min_ and CT_max_) in a subset of lines from the DGRP. CT_min_ values across the DRGP lines varied considerably more than CT_max_ (Figures 1A,B). CT_min_ ranged from 0.81 to 8.55°C for males and from 2.29 to 8.64°C for females, while CT_max_ ranged from 38.63 to 40.72°C for males and from 38.51 to 40.80°C for females (Supplementary Table 1). The sex of the flies did not affect CT_min_ (*p* = 0.39), but it did affect CT_max_ (*p* < 0.001), with the males being slightly more heat tolerant than the females. However, the effect size was small (effect size: 0.13°C). The interaction of sex and line was also significant for both CT_min_ and CT_max_ (*p* < 0.01 and *p* < 0.001, respectively). Within each phenotype, values were significantly correlated across sexes (CT_min_, *r* = 0.85, *p*-value < 0.001; CT_max_, *r* = 0.52, *p*-value < 0.001; Supplementary Figures 2A,B). The sex-averaged CT_min_ and CT_max_ values were not significantly correlated across lines (*r* = 0.06, *p*-value = 0.54; Figure 1C).

We tested for trade-offs associated with thermal tolerance by comparing CT_min_ and CT_max_ with previously collected lifespan and fecundity data (Durham et al., 2014); however, we found no evidence of trade-offs among these traits, as neither thermal tolerance measurement was correlated with longevity or fecundity (Supplementary Table 2). DGRP lines have variable infection status by *Wolbachia pipientis*, a ubiquitous endosymbiont in insects that can significantly modulate physiology (Werren, 1997). Within the lines studied, we found no evidence that *Wolbachia* infection impacts CT_min_ or CT_max_ (*p* = 0.07 and *p* = 0.19, respectively). We also performed correlations between our data and other measures of thermal tolerance, and we found no correlation among our results and chill coma recovery time (Mackay et al., 2012), measures of thermal plasticity (i.e., rapid cold hardening and survival from cold; Gerken et al., 2015), cumulative cold tolerance (Teets and Hahn, 2018), and heat knockdown time (Rohde et al., 2016; Supplementary Table 2). Ørsted et al. (2018) measured CT_min_ in males reared at 26°C (using a ramping method of 0.1°C min^–1^), and we found significant correlations among their CT_min_ values with our CT_min_ values for males and females (*r* = 0.60, *p*-value < 0.01 and *r* = 0.61, *p*-value < 0.01, respectively; Supplementary Table 2). As in our study, Rolandi et al. (2018) also measured CT_max_ using a ramping method (0.25°C min^–1^). We found significant correlations between their CT_max_ values for males with our CT_max_ values for males and females (*r* = 0.59, *p*-value < 0.01 and *r* = 0.55, *p*-value < 0.01, respectively; Supplementary Table 2).

### Genetic Architecture of Thermal Limits

The estimated broad sense heritability (*H*^2^) for CT_max_ was 0.29 and for CT_min_ was 0.25.

**FIGURE 2:**
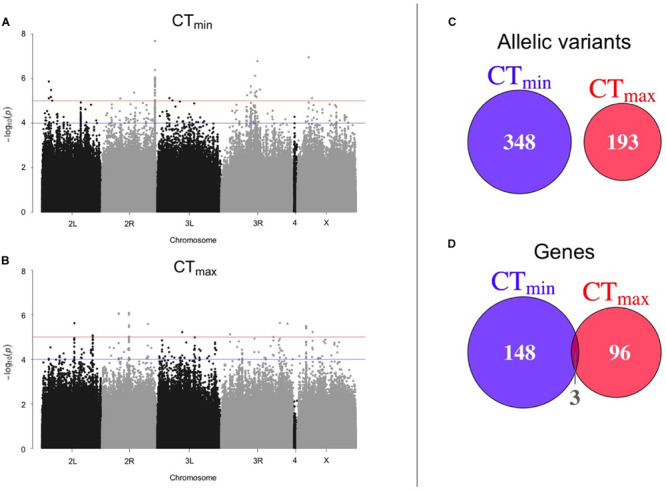
Results of GWAS to identify polymorphisms associated with thermal tolerance. Manhattan plots of the results from the GWAS for **(A)** CT_min_ and **(B)** CT_max_. The blue line corresponds to *p*-value < 1E-4 and the red line corresponds to *p*-value < 1E-5. The overlap of **(C)** allelic variants and **(D)** unique genes between CT_min_ and CT_max_ is also shown.

**FIGURE 3:**
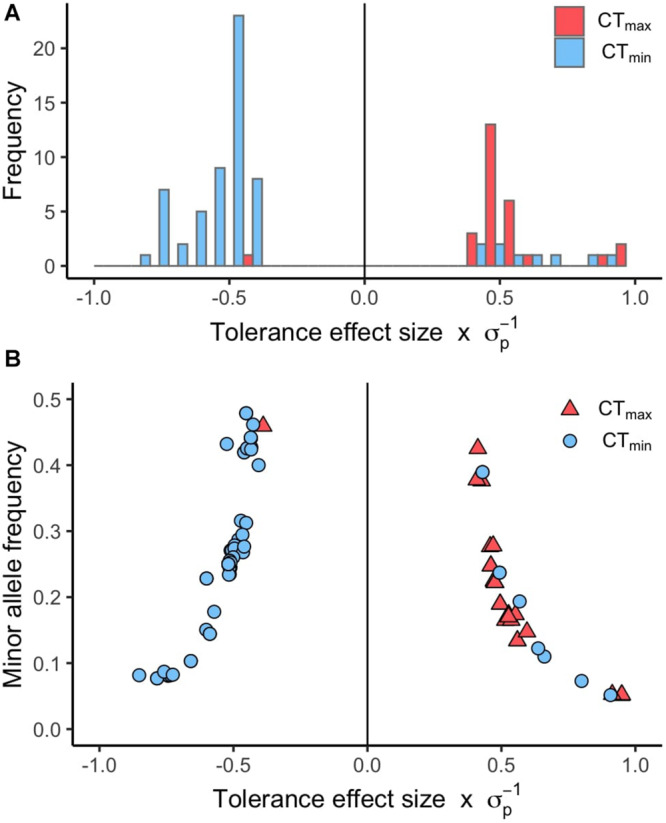
Distribution of effect sizes for SNPs associated with CT_min_ and CT_max_. **(A)** Histogram of the tolerance effect size scaled by the phenotypic standard deviation (σ_p_) for the same allelic variants. **(B)** Scatter plot of the minor allele frequency versus the tolerance effect size scaled by the phenotypic standard deviation (σ_p_) for the allelic variants associated with CT_min_ and CT_max_ (*p*-value < 1E-5).

Using available genomic data for the DGRP, we identified genetic polymorphisms associated with CT_min_ and CT_max_. For the 99 lines measured, more than 1.9 million variants were analyzed (mostly SNPs), and we found ∼550 unique allelic variants associated with these traits (*p*-value threshold of 1E-4; Supplementary Table 3). We identified 348 allelic variants (319 SNPs, 15 deletions, 13 insertions, and 1 MNP) significantly associated with CT_min_, and 193 allelic variants (173 SNPs, 9 deletions, 8 insertions, and 3 MNPs) with CT_max_ (Table 1). Polymorphisms associated with CT_min_ and CT_max_ were identified on all chromosomes (Figures 2A,B). CT_min_ and CT_max_ did not share any allelic variants (Figure 2C). Among these allelic variants, 262 mapped to 151 unique genes for CT_min_, and 169 mapped to 99 unique genes for CT_max_. Three genes (*iab8*, *Btk29A* and *Sp1*) were common between both traits (Figure 2D). From all the genes associated with the allelic variants, 8% of the CT_min_ and 9% of the CT_max_ genes encode transcription factors (Table 1), including one of the genes common to both traits (*Sp1*). Most of the allelic variants significantly associated with both traits were located in introns (55% for CT_min_ and 71% for CT_max_; Supplementary Table 4). The distribution of the direction of effect sizes differed between SNPs that underlined CT_min_ vs. SNPs that underlined CT_max_ (Figure 3A; Kolmogorov–Smirnov test, *D* = 0.87, *p*-value < 1E-9), such that the CT_min_-associated alleles that were most common in the DGRP population caused individuals to have higher CT_min_ (i.e., worse cold tolerance; Figure 3B), whereas the CT_max_-associated alleles that were most common in the population overwhelmingly caused individuals to have higher CT_max_ (i.e., better heat tolerance; Figure 3B). Additionally, there was a negative exponential relationship between effect size and minor allele frequency for both CT_min_- and CT_max_-associated SNPs (Figure 3B).

**TABLE 1:** Overlap between the CT_min_ and CT_max_ SNPs and expression data.

GWAS	Relaxed SNPs	Strict SNPs	Unique genes	DEGs	TFs	DE TFs
CT_min_	348	53	151	72	12	3
CT_max_	193	21	99	59	9	9

*Counts of Relaxed SNPs (*p*-value < 1E-4), Strict SNPs (*p*-value < 1E-5), Unique genes (based on relaxed SNPs), differentially expressed genes (DEGs; FDR < 0.01), transcription factors (TFs), and differentially expressed transcription factors (DE TFs).*

There is some evidence that low abundance alleles can be underpowered in the DGRP (Ivanov et al., 2015), so as an alternative to the variant-based GWAS performed above, we also conducted gene-based GWAS. However, in the gene-based GWAS analysis, almost no genes were detected as significant using the *p*-value threshold of 1E-4 (three for CT_min_ and one for CT_max_; Supplementary Table 5). Thus, for the remainder of the paper, we will focus on the results of the variant-based GWAS described above.

### Differential Gene Expression Following Acute Thermal Exposures

**FIGURE 4:**
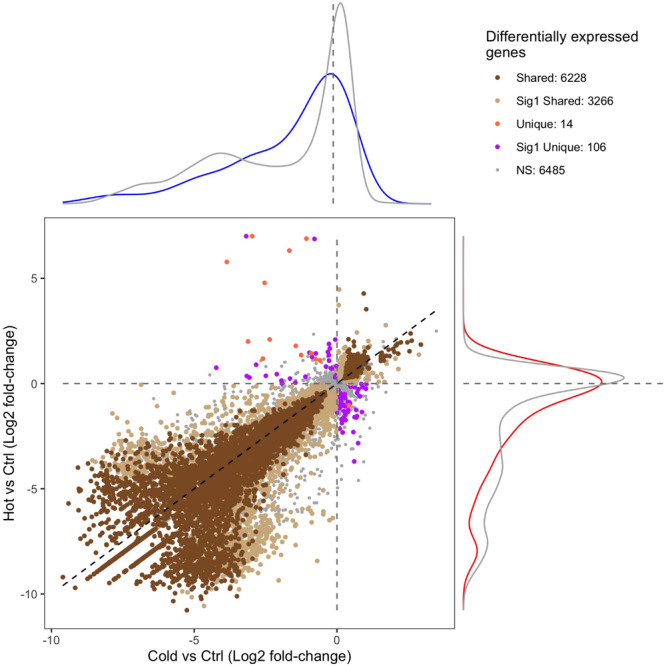
Scatter plot of the gene expression log_2_ fold-change of heat-shock (37°C) and cold-shock (4°C) relative to the 25°C control. “Shared” DEGs indicate a significant expression relative fold-change in the same direction in both heat and cold shock. “Sig1 Shared” genes indicate that the relative fold-change is the same under heat shock and cold shock, but only significantly differentially expressed in one condition. “Unique” DEGs indicate that the genes were differentially expressed in each condition but in opposite directions. “Sig1 Unique” genes indicate that the genes are expressed in opposite directions relative to the control, but only significantly so in one condition. “NS” indicates genes that are not differentially expressed in both heat shock and cold shock. The density plots surrounding the figure indicate the density of the expression of genes that are associated with CT_min_ (top; blue) or CT_max_ (right; red) relative to the log_2_ fold-change expression of all other genes (gray).

RNA-seq and differential expression analysis were used to determine the gene expression responses of the Canton-S strain of *D. melanogaster* under ramped cold shock and heat shock conditions. In total, 15,844 genes were expressed across all treatment groups. Principal component analysis (PCA) of expressed genes showed clustering of replicates for each condition (Supplementary Figure 3). Pairwise comparison of cold shock and heat shock to controls revealed a large number of significantly differentially expressed genes (*p*-adj. < 0.05, fold-change > 2; Figure 4). Among the differentially expressed genes, more were downregulated (5,126 in cold shock and 6,241 in heat shock) and fewer were upregulated relative to controls (1,826 in cold shock and 2,314 in heat shock). The direction of regulation for most differentially expressed genes was consistent across treatments, with 6,081 genes changing similarly in both magnitude and direction in response to cold and heat shock conditions (Figure 4).

### Integration of Transcriptomics and GWAS

Of the 151 unique genes found for CT_min_, 72 (47.7%) were differentially expressed under cold shock. Of the 99 unique genes associated with CT_max_, 59 (59.6%) were differentially expressed under heat shock. The distribution of log-fold change expression of heat shock versus control for the GWAS candidates associated with CT_max_ significantly differed from the distribution of all other genes under heat shock (Figure 4; *p*-value < 0.01). For the genes associated with CT_min_, there was a similar trend, but the log-fold change distribution of those genes only marginally differed from the background expression of all other genes (Figure 4; *p*-value = 0.062).

### Overrepresentation Analysis

**FIGURE 5:**
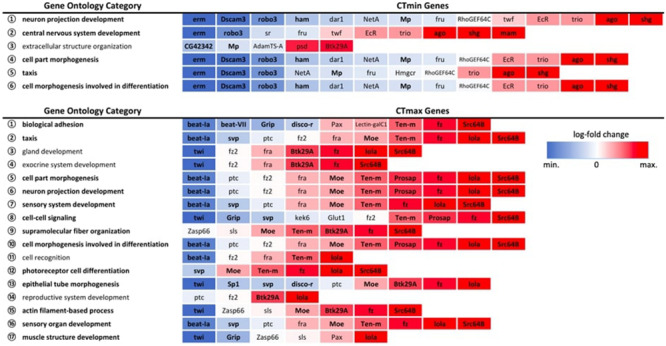
Expression patterns of GWAS genes within significantly overrepresented GO biological process categories. All expression patterns are expressed relative to the 25°C control. Bolded categories indicate those also over-represented in the full DEG dataset for the corresponding temperature extreme. Bolded genes indicate significant differential gene expression at an FDR < 0.01.

Among the GWAS candidates, we identified six overrepresented GO biological process categories for CT_min_ and 17 categories for CT_max_ that met the FDR cut-off of 0.05 (Figure 5). For both traits we found many enriched GO terms related to development, differentiation, and morphogenesis. Among the differentially expressed genes from the RNA-seq experiments, we identified 99 enriched GO biological process categories for cold shock and 113 enriched categories for heat shock (Supplementary Table 6). Many of these categories were also related to development and differentiation. There was overlap between the enriched categories for GWAS and gene expression data. Five of the six categories enriched among genes associated with CT_min_ were also significantly enriched among genes differentially expressed under cold shock, and 13 of the 17 categories identified for CT_max_ were also enriched under heat shock (Figure 5).

## Discussion

Here, we characterized the genetic architecture of thermal tolerance and identified candidate genes that contribute to both dynamic responses to thermal stress and preparative processes that enhance stress resistance. Our study suggests that GWAS candidates are involved in both dynamic stress responses and preparative processes that influence the condition of the insect at the time of thermal stress. Together, our results indicate diverse functions for genes involved in thermal tolerance and allow us to generate new hypotheses for the genetic basis of thermal tolerance. Below, we discuss the genetic architecture of thermal tolerance in general, followed by a discussion of our three specific hypotheses to test the relative contribution of dynamic and preparative processes in shaping thermal tolerance.

### Genetic Architecture of Thermal Tolerance

While several studies have separately assessed the genetic basis of cold and heat tolerance, here we measured both CT_min_ and CT_max_ across 100 lines of the DGRP. We found variation in both measures, although CT_min_ varied considerably more than CT_max_ (Figure 1). This pattern of variation in upper and lower thermal limits is also seen across species and populations with distinct geographic ranges, for both latitudinal and altitudinal gradients (e.g., Gaston and Chown, 1999; Addo-Bediako et al., 2000; Chown, 2001; Hoffmann et al., 2002; Nyamukondiwa et al., 2011; Sunday et al., 2011, 2019; Kellermann et al., 2012a, b). These patterns of variation in thermal limits, both within and across species, likely reflect stronger latitudinal and interannual variation in winter conditions relative to summer conditions (Williams et al., 2015). In addition, our results are consistent with previous studies indicating that upper and lower limits have distinct underlying mechanisms (e.g., Chown, 2001; Nyamukondiwa et al., 2011), as we found no phenotypic correlation between CT_min_ and CT_max_ across lines.

The variation in both CT_min_ and CT_max_ had a strong genetic component. Broad sense heritability was high for both CT_max_ (*H*^2^ = 0.29) and CT_min_ (*H*^2^ = 0.25), which is consistent with previous heritability estimates for thermal responses in the DGRP. Our heritability estimate for CT_max_ was higher than a previous estimate for a smaller subset of the DGRP population (*H*^2^ = 0.14, Rolandi et al., 2018). For CT_min_, heritability was within the range observed by Gerken et al. (2015) for acute and chronic cold exposure (*H*^2^ = 0.15 and 0.44, respectively). The strong heritability for both traits suggests high evolutionary capacity for thermal tolerance in the mid-latitude population from which the DGRP was derived.

While variation in CT_min_ and CT_max_ was explained by distinct allelic variants, some variants mapped to the same genes, suggesting that some genes can affect both heat and cold tolerance. Of the 247 total unique genes, three genes were common to both traits: *iab-8* (a non-coding regulatory RNA), *Btk29A* (a tyrosine kinase involved in cellularization and morphogenesis), and *Sp1* (a zinc finger transcription factor involved in ventral thoracic appendage specification, leg growth and in the development of type-II neuroblasts). At the gene level, there were also some candidate genes in common between our study and previous work. The gene CG42673 was associated with CT_max_ in this study and also with chill coma recovery time in Mackay et al. (2012). CG42673 is a putative nitric-oxide synthase binding protein, and while nitric oxide has not been linked to thermal tolerance in insects, nitric oxide is an important mediator of both heat and cold tolerance in plants (Parankusam et al., 2017; Costa-Broseta et al., 2018). In both our study and in Ørsted et al. (2018), *Mur89F* was associated with CT_min_, and this gene is involved in chitin metabolic process and extracellular matrix.

Several patterns in our data suggest that thermal tolerance, especially heat tolerance, is an important component of organismal fitness in nature. Alleles that enhance heat tolerance (i.e., raise CT_max_) were more common in the DGRP population than alleles that impair heat tolerance (Figure 3A). However, the pattern is opposite for cold tolerance; most alleles that improve cold tolerance (i.e., lower CT_min_) were relatively infrequent in the population (Figure 3A). These results were counter to our expectations, since there is generally stronger latitudinal variation in cold tolerance than heat tolerance (see above). However, intraspecific latitudinal clines for heat tolerance have been observed in *D. melanogaster* (Hoffmann et al., 2002; Sgrò et al., 2010), indicating that there is selection for heat tolerance in this species. In the case of the DGRP, which originates in mid-latitude North Carolina, selection for cold tolerance may be lower than for heat tolerance, which could explain the relative rarity of alleles that improve cold tolerance. Alternatively, alleles that improve cold tolerance may have negative effects on other fitness-related traits, which would prevent these alleles from increasing in frequency in the population. Further, some polymorphisms in *D. melanogaster* oscillate in allele frequency across seasons (Bergland et al., 2014), so depending on when the DGRP was collected (presumably summer, although exact details are not provided in Mackay et al., 2012), cold tolerance alleles might be less common in the panel. Previously, a similar pattern was shown for oxidative stress resistance in the DGRP – i.e., most alleles that improve oxidative stress response are present at low frequency in the population (Weber et al., 2012). Finally, our study did not address plasticity in thermal tolerance, which may be an important means of response to cold challenges. Thus, the rarity of alleles improving basal cold tolerance may not be relevant in this population if the population possesses alleles for thermal plasticity. However, regardless of the reason for these results, these allele frequency patterns between heat and cold tolerance are not random and suggest that different forces may be affecting patterns of standing genetic variation for the two traits despite their similar overall heritabilities.

We also observed a negative exponential relationship between effect sizes and minor allele frequencies for SNPs that underlie both CT_min_ and CT_max_ (Figure 3B). Overwhelmingly, the SNPs that have the greatest effect sizes in both directions are present at the lowest frequencies in the population. Many other studies have reported this same pattern for genetic polymorphisms that underlie a wide range of different traits in the DGRP, including oxidative stress resistance, startle response, starvation resistance, chill coma recovery time, and position effect variegation (Mackay et al., 2012; Weber et al., 2012; Kelsey and Clark, 2017). Overall, this pattern suggests that large-effect alleles that underlie CT_min_ or CT_max_ have undergone selection, either to increase the frequency of large-effect alleles in the population by positive selection (Barton and Keightley, 2002), thus driving the alternative allele to low frequency, or to remove large-effect alleles from the population by purifying selection (Keightley and Lynch, 2003).

Some of the genes underlying variation in CT_min_ and CT_max_ in the DGRP are also likely important for temperature adaptation in natural populations. Previous work by Fabian et al. (2012) and Bergland et al. (2016) used F_ST_ outlier analyses to identify loci across the genome that are likely to be undergoing adaptive divergence among populations of *D. melanogaster* that inhabit different thermal environments in eastern North America and eastern Australia. Among the 109 candidate genes that showed convergent patterns of clinal latitudinal differentiation in North America and Australia (Fabian et al., 2012; Bergland et al., 2016), seven of these genes were associated with CT_max_ and one was associated with CT_min_ in the DGRP. The clinal genes associated with CT_max_ were *beat-VII*, *dpr8*, *CG33970*, *CG42322*, *Tsp66E*, *A2bp1*, and *Moe*, and the sole clinal gene associated with CT_min_ was *fru*. Most of these clinal CT_max_ genes also changed expression following heat stress: three genes were downregulated (*beat-VII*, *dpr8*, and *CG42322*), two genes were upregulated (*Tsp66E* and *Moe*), and two genes (*CG33970* and *A2bp1*) were not differentially expressed (Supplementary Table 7). These genes are involved in a myriad of cellular and developmental processes, but a general theme is the potential role of neuronal processes in the thermal adaptation of heat tolerance (see below for additional discussion on the nervous system). It is also interesting to note the potential role of regulatory genes in thermal adaptation. Specifically, the CT_max_ clinal gene *A2bp1* is an RNA-binding Fox protein that regulates transcription and mRNA translation (Usha and Shashidhara, 2010; Carreira-Rosario et al., 2016), and the sole CT_min_ clinal gene *fru* is a zinc finger transcription factor known to be a regulator of transcriptional activity of many genes across various tissues (Sato and Yamamoto, 2020). While these genes did not dynamically respond to heat or cold stress (Supplementary Table 7), they may be important for setting up the developmental and/or cellular contexts in which stress responses operate.

### H1: Genes Associated With Thermal Tolerance Are Involved in Dynamic Stress Responses

Thermal tolerance is shaped by a combination of preparative processes that improve stress resistance and dynamic changes in gene expression and activity that occur during and after stress. Dynamic changes in gene expression are well-established responses to thermal stress, and here we observed sweeping changes in gene expression in response to temperature change. Ramping at 0.25°C min^–1^ toward both temperature extremes elicited transcriptome-wide gene expression changes, with 43.9% and 54.0% of detected genes differentially expressed under cold and heat shock, respectively. These values are substantially larger than those reported in other studies, which range from minimal transcriptional response to ∼15% of the transcriptome, depending on the methodology used (Zhou et al., 2012; Telonis-Scott et al., 2013; von Heckel et al., 2016; Königer and Grath, 2018).

By pairing GWAS and RNA-seq using similar ramping methodologies, we can assess the extent to which GWAS candidates are directly involved in dynamic stress responses. GWAS-associated CT_max_ genes were significantly more temperature-responsive than the transcriptome at large and CT_min_ genes were marginally so (Figure 4), suggesting that genes associated with thermal tolerance are involved in the dynamic response to thermal stress. This congruence was also mirrored in the intersection of overrepresented GO terms in the two datasets, with five of six categories overrepresented in the CT_min_ GWAS also enriched among genes of cold shock response, and 13 of 17 CT_max_ categories shared with the heat shock response (Figure 5).

Despite a lower total number of genes identified that underlie CT_max,_ the total set of overrepresented biological process categories was more diverse than for CT_min_ and included cell signaling, muscle structure and function, and the sensory system (Figure 5). This may indicate a stronger role of active defensive responses in setting CT_max_. At the individual gene level, the majority of top GWAS hits for CT_max_ were thermally responsive (Table 1) and were similarly diverse in function, including genes involved in neuropeptide signaling, mRNA processing, and protein dephosphorylation and catabolism. In contrast to the cold response, which included a mix of downregulated and upregulated genes, the majority of CT_max_ GWAS hits found within thermally responsive categories were upregulated under heat ramp conditions, suggesting that they are involved in active protection from or in response to heat damage (Figure 4). Thus, although the magnitude of phenotypic variation in CT_max_ is substantially lower than that of CT_min_, standing genetic variation may be mediated via a wider range of defensive mechanisms, each of small effect.

Interestingly, the GWAS analysis did not indicate a role for the genes most commonly associated with thermal tolerance in experimental work. Much of the early literature on thermal limits focused on the effects of copy number and regulatory control of the heat shock protein (*hsp*) genes (Welte et al., 1993; Feder et al., 1996), and natural selection may also affect *hsp* allele frequencies (e.g., *hsp70*; Bettencourt et al., 2002). However, neither *hsp* genes nor their regulatory factors (e.g., hsf-1) were identified from the GWAS analysis as causal drivers of variation for either heat or cold limits within the DGRP. These *hsp* genes and their regulatory factors (hsf) did increase in expression in response to both cold and heat shock, so while these canonical stress genes had clear roles in dynamic stress responses, polymorphisms in these genes were not associated with thermal limits in the DGRP. For cold tolerance, *Frost* (*Fst*) is commonly upregulated in response to cold acclimation and during recovery from cold stress (Goto, 2001; Qin et al., 2005; Sinclair et al., 2007; Colinet et al., 2010), and it is also located within a QTL for chill coma recovery time (Morgan and Mackay, 2006). In our study, *Frost* was not associated with variation for thermal limits. Further, it was not differentially expressed following cold shock but was upregulated after the heat shock. The lack of upregulation during cold stress is likely due to flies being sampled at 4°C, as *Frost* expression typically only increases during recovery from cold stress (Bing et al., 2012). The unexpected upregulation during a heat ramp suggests that *Frost* may be involved in heat stress, in addition to its role for cold and desiccation stress (Sinclair et al., 2007). Likewise, *Starvin* (*stv*), a poorly studied gene that is strongly upregulated during recovery from cold stress (Colinet and Hoffmann, 2010), was not associated with thermal tolerance but was upregulated after heat shock in this study.

### H2: Genes Associated With Thermal Tolerance Have Regulatory Functions

Changes in gene transcription are one of the primary cellular responses to cold and heat stress in this and other studies (Gasch et al., 2000; Leemans et al., 2000; Gracey, 2007; Lockwood et al., 2010; Brown et al., 2014; Sørensen et al., 2016). Moreover, there is a direct connection between whole-organism stress tolerance and the ability to transcriptionally respond to stress, as organisms with limited transcriptional stress responses have lower survival following exposure to stress (Welte et al., 1993; Feder et al., 1996; Hofmann et al., 2000). Therefore, we expected to find candidate genes for thermal tolerance that have gene regulatory functions, such as transcription factors. Polymorphisms in regulatory genes or genomic regions may modify transcriptional responses to thermal stress, and thereby confer phenotypic differences in whole-organism thermal tolerance (Zatsepina et al., 2001; Bettencourt et al., 2002; Lerman et al., 2003). We report evidence for this potential regulatory effect among the SNPs that underlie both heat and cold tolerance, but consistent with the stronger pattern of upregulation in the dynamic response to heat, our results suggest a larger role of transcription factors in driving genetically based variation in CT_max_ than in CT_min_.

Overall, there were nine CT_max_-associated transcription factor genes (Table 1), and all were differentially expressed in response to heat stress (Supplementary Table 7). Indeed, the top four SNPs that were associated with CT_max_ (lowest *p-*value; Supplementary Table 3) were in two genes that encode transcription factors, *Oaz* and *lola*. *Oaz* encodes a transcription factor known to be involved in spiracle development (Krattinger et al., 2007), and thus may mediate developmental mechanisms that impact heat tolerance, especially since spiracles facilitate gas exchange and failings of aerobic respiration may set upper thermal limits (Dahlhoff and Somero, 1993; Pörtner, 2002). *Oaz* may also be involved in regulating the dynamic transcriptional response to heat stress, as it was the most strongly downregulated transcription factor following heat stress (Supplementary Table 7). *lola* is involved in a diverse array of cellular and developmental processes (Thurmond et al., 2019), and is represented among several GO categories in Figure 5. All four of the top CT_max_ SNPs lie in introns of the coding sequences of *Oaz* or *lola*, suggesting that these polymorphisms influence gene regulation (Bicknell et al., 2012). Indeed, in the case of *lola* both mutations lie in a region that is a putative transcription factor binding site. While previous work also showed that these two genes respond to heat stress in *D. melanogaster* (Brown et al., 2014), to our knowledge no previous studies have identified a functional role for these genes in heat tolerance. Another notable CT_max_-associated SNP lies in two overlapping genes that encode the transcription factors HmgD and HmgZ; the CT_max_ SNP lies in the 5′ UTR intron of *HmgZ* and in the putative upstream regulatory region of *HmgD*. These genes encode proteins that belong to the family of high mobility group box transcription factors that are known to facilitate gene transcription by promoting DNA structural flexibility via chromatin remodeling (Štros, 2010), and both of these genes were differentially expressed following heat stress (Supplementary Table 7). Interestingly, high mobility group proteins have previously been reported to show expression patterns that track environmental temperature in killifish, *Austrofundulus limnaeus* (Podrabsky and Somero, 2004). Thus, the regulation of gene transcription may be a key aspect of heat tolerance in *D. melanogaster*.

The genetic architecture of cold tolerance also included genetic variation in transcription factor genes, but most of the CT_min_-associated transcription factor genes did not change expression following cold stress, suggesting a qualitative difference in the role of transcription factors in heat vs. cold tolerance. While there were 12 transcription factor genes with significant associations with CT_min_ (Table 1), only three of these genes changed expression following cold stress (Supplementary Table 7). Importantly, one of the top SNPs in association with CT_min_ lies in the gene *blistered* (*bs*) (Supplementary Table 3). *bs* encodes a transcription factor known to be involved in a variety of developmental processes, including wing morphogenesis (Dworkin and Gibson, 2006), tracheal development (Affolter et al., 1994), and neural system development (Donlea et al., 2009; Thran et al., 2013). Similar to *lola*, the thermal tolerance SNP in *bs* lies in an intron with a transcription factor binding site; however, in contrast to *lola* and the other CT_max_-associated transcription factor genes, *bs* did not change expression in response to cold shock (Supplementary Table 7).

One of the three genes associated with both CT_min_ and CT_max_, the long non-coding RNA (lncRNA) *iab-8*, was downregulated in both cold and heat shock. LncRNAs have been implicated in a range of biological processes and are emerging as key regulators of gene expression at transcriptional and post-transcriptional levels (Li et al., 2019). *In vivo* studies of lncRNAs revealed that dysregulated expression of lncRNAs in *Drosophila* may result in poor stress resistance (Lakhotia et al., 2012). The *iab-8* lncRNA, expressed in the embryonic abdominal segment 8, represses the expression of the *abd-A* gene in the posterior central nervous system (Li et al., 2019). The *abd-A* gene is linked with neural system development (Bello et al., 2003; Cenci and Gould, 2005), and as discussed below, the nervous system likely plays an important role in thermal tolerance.

### H3: Genes Involved in Thermal Tolerance Affect the Developmental and Structural Context

Thermal tolerance occurs within a developmental and structural context, making physiological systems more or less resistant to temperature challenges (González-Tokman et al., 2020). Thus, genes associated with thermal tolerance may do so by altering the physiological condition of the organism at the time of thermal stress. Because these genes alter baseline preparedness prior to application of cold or heat, the expression of these genes may not directly respond to temperature changes. Moreover, we would expect their biological function to be concentrated in processes underlying thermal stability of physiological functions, such as the central and peripheral nervous system, cell membrane composition, and proteome stability (Cossins and Prosser, 1978; Gu and Hilser, 2009; Cooper et al., 2012; Fields et al., 2015; MacMillan et al., 2015a; Willot et al., 2017).

Our results suggest that although segregating variation in both heat and cold tolerance is likely to include some structural effects, preparative processes that enhance thermal stress resistance appear to play a stronger role for CT_min_. Fully half of the CT_min_ GWAS genes did not change significantly in expression in response to cold exposure, regardless of the significance cutoff used (Table 1). These included a cluster of functionally related genes (*dar1, fru, NetA, RhoGEF64C, trio, twf)* involved in nervous system development, which was reflected in overrepresentation of the nervous system and cell morphogenesis and differentiation GO categories in the CT_min_ GWAS gene set. Neuronal failure operationally defines both CT_min_ and CT_max_ (Andersen et al., 2018; Andersen and Overgaard, 2019; Jørgensen et al., 2019), and dynamic stabilization of the neuromuscular circuit under temperature stress is a likely mechanism for altering thermal limits. Indeed, previous investigation of the genetic architecture of cold hardiness and electrophysiological analyses of the rapid hardening response both suggest an important role for stabilization of ion channels and cytoskeletal structures supporting the synapse and neuromuscular junction (Klose and Robertson, 2004; Robertson and Money, 2012; Freda et al., 2017). Aside from genes involved in neural morphogenesis, the GO term extracellular structure organization was overrepresented among cold tolerance genes, and this was the only overrepresented category that did not overlap with the differential expression categories. The glial-derived extracellular matrix is integrally involved in development, stabilization and plasticity of neuronal synapses, and is involved in promoting cell survival, facilitating repair, and maintaining synaptic current amplitude under stress conditions (Dityatev et al., 2010; Faissner et al., 2010; Wang et al., 2018). Together, these results suggest that stabilization of nervous function is an important component of cold tolerance, which is consistent with recent physiological literature (reviewed by Overgaard and MacMillan, 2017).

For CT_max_, more of the GWAS candidate genes were differentially expressed, especially when considering the strongest candidates (Supplementary Table 7). Relatively few of the non-responsive genes were found within overrepresented categories, with a range of only 0–3 included in each of the 17 enriched CT_max_ GO categories (Figure 5). The few genes that consistently appeared in overrepresented categories, *fra, fz2* and *ptc*, are also functionally associated with the nervous system, including axon and dendrite guidance and synapse organization. Spreading depolarization of the central nervous system (triggered by failure to maintain ion gradients between the intra- and extracellular compartments) is linked with heat tolerance across *Drosophila* species (Jørgensen et al., 2019), indicating that neuronal failure is an important component of heat tolerance in addition to cold tolerance. An additional set of three genes, *Pax, sls* and *Zasp66*, co-occurred in several associated categories of muscle structure and development (Figure 5). Depolarization of muscles has been associated with lower thermal limits (e.g., MacMillan et al., 2014, 2015b) but to our knowledge has not been linked to upper limits.

## Conclusion

Several studies have separately assessed the genetic architecture and plastic transcriptional responses to thermal stress, but the extent to which genes associated with thermal tolerance are involved in preparative and dynamic stress responses has not been assessed. Here, we show that genes associated with variation in thermal tolerance, identified via GWAS, included differentially expressed genes directly involved in the dynamic stress response, as well as a number of transcription factors that likely regulate these processes. However, while GWAS candidate genes were more likely to be differentially expressed, genes commonly associated with thermal stress, such as heat shock proteins (*hsp*), were not identified among GWAS genes. These core stress genes tend to be highly conserved, so it is likely that these genes have little genetic variation, especially in functional regions. In addition, consistent with previous studies (e.g., Sørensen et al., 2005; Telonis-Scott et al., 2013; MacMillan et al., 2016), most of the differentially expressed genes were downregulated for both hot and cold stresses. This result suggests an important role for shutting down certain biological processes during stress, and future studies should address these processes that are incompatible with stress tolerance, in addition to the well-studied protective pathways that are activated by stress.

A noteworthy finding of our study is a prominent role for genes involved in preparatory physiological processes that influence the condition of the organism at the time of thermal stress. While we did not observe an abundance of genes involved in processes commonly associated with preparation for thermal stress, such as cell membrane, circadian function, and immune response (Sinensky, 1974; Koštál, 2010; Teets and Hahn, 2018), we found a strong representation of nervous system processes for both CT_min_ and CT_max_ GWAS genes. Many of these genes have defined roles in development or morphogenesis, suggesting that developmental processes can influence thermal tolerance later in life, or that some of these genes are co-opted for thermal tolerance later in life. Future validation with other tools, such as RNA interference and transgenic overexpression, can clarify the precise role of these genes in thermal tolerance.

While all three of our hypotheses were supported for both cold and heat tolerance, genes associated with upper thermal limits tended to be more involved in dynamic stress responses than those associated with cold tolerance. Furthermore, some genes associated with thermal tolerance appear to play multiple roles, highlighting that our three hypotheses are not mutually exclusive and that some genes likely have pleiotropic roles to shape thermal tolerance. Both upper and lower thermal limits had a strong genetic component, but the genetic signatures for these traits were largely distinct, with no overlapping SNPs and only three overlapping genes. Thus, heat and cold tolerance involve distinct molecular processes and can likely independently involve in response to changing environmental conditions.

## Data Availability Statement

All datasets generated for this study are included in the article/Supplementary Material. Sequencing data have been deposited into the NCBI SRA database with accession Bioproject PRJNA612361.

## Author Contributions

NT, BL, SC, JW, and HA acquired the financial support for the project leading to this publication. NT, BL, and SC conceived and designed the study. ML, DA, LU, NJ, MW, EM, BP, and KB performed the experiments and data collection. ML, DA, TO’L, SF, BL, NT, and SC analyzed the data. ML, DA, TO’L, SF, BL, NT, and SC wrote the manuscript. All authors approved the final version of the manuscript.

## Conflict of Interest

The authors declare that the research was conducted in the absence of any commercial or financial relationships that could be construed as a potential conflict of interest.

## Footnotes

1 http://dgrp2.gnets.ncsu.edu
