# Supplementary material for: Integrating GWAS and Transcriptomics to Identify the Molecular Underpinnings of Thermal Stress Responses in Drosophila melanogaster
Source: Front Genet. 2020 Jun 23;11:658. doi: 10.3389/fgene.2020.00658 (PMC7324644; doi:10.3389/fgene.2020.00658)
Supplement: Supplementary file 2 [file Table_2.docx]

**Supplementary Table 2.** **Correlations between CT_min_ and CT_max_ from this study and life-history traits and thermal tolerance measures found in literature.** Bold values denote statistical significance at the p-value < 0.05 level.

|  |  |  | **CT_min_** | | | | **CT_max_** | | | |
| --- | --- | --- | --- | --- | --- | --- | --- | --- | --- | --- |
|  |  |  | **Males** | | **Females** | | **Males** | | **Females** | |
|  |  |  | **r** | **p** | **r** | **p** | **r** | **p** | **r** | **p** |
| Durham et al., 2014 | **Lifespan** | **Females** |  |  | -0.03 | 0.913 |  |  | 0.13 | 0.579 |
|  | **Fecundity** |  |  |  | 0.03 | 0.9 |  |  | 0.31 | 0.179 |
| Mackay et al., 2012 | **Chill coma recovery** | **Males** | 0.13 | 0.584 |  |  | -0.31 | 0.177 |  |  |
|  |  | **Females** |  |  | 0.04 | 0.862 |  |  | -0.32 | 0.155 |
| Gerken et al., 2015 | **Rapid cold hardening** | **Males** | 0.01 | 0.968 |  |  |  |  |  |  |
|  |  | **Females** |  |  | -0.07 | 0.764 |  |  |  |  |
|  | **Chronic cold exposure** | **Males** | 0.02 | 0.945 |  |  |  |  |  |  |
|  |  | **Females** |  |  | 0.11 | 0.634 |  |  |  |  |
|  | **Acute cold exposure** | **Males** | 0.18 | 0.442 |  |  |  |  |  |  |
|  |  | **Females** |  |  | 0.05 | 0.827 |  |  |  |  |
| Teets and Hahn, 2018 | **Cumulative cold tolerance** | **Females** |  |  | -0.19 | 0.419 |  |  |  |  |
| Rohde et al., 2016 | **Heat knockdown time** | **Females** |  |  |  |  |  |  | 0.12 | 0.616 |
| Ørsted et al., 2018 | **CT_min_** | **Males** | **0.6** | **0.004** | **0.61** | **0.003** |  |  |  |  |
| Rolandi et al., 2018 | **CT_max_** | **Males** |  |  |  |  | **0.59** | **0.005** | **0.55** | **0.01** |
|  |  | **Females** |  |  |  |  | 0.41 | 0.068 | 0.26 | 0.247 |
