# Supplementary material for: Integrating GWAS and Transcriptomics to Identify the Molecular Underpinnings of Thermal Stress Responses in Drosophila melanogaster
Source: Front Genet. 2020 Jun 23;11:658. doi: 10.3389/fgene.2020.00658 (PMC7324644; doi:10.3389/fgene.2020.00658)
Supplement: Supplementary file 4 [file Table_4.docx]

**Supplementary Table 4. Counts of number of SNPs associated with CT_min_ and CT_max_ (p-value < 1E-4) that lie within each genomic feature group.**

| **Feature** | **CT_min_** | **CT_max_** |
| --- | --- | --- |
| Intron | 171 | 152 |
| Downstream | 40 | 22 |
| Upstream | 35 | 16 |
| Synonymous coding | 31 | 13 |
| 3’ UTR | 11 | 3 |
| Non-synonymous coding | 10 | 7 |
| 5’ UTR | 8 | 2 |
| Exon | 4 | 0 |
| Start gained | 1 | 0 |
