# Supplementary figures and images for: Integrating GWAS and Transcriptomics to Identify the Molecular Underpinnings of Thermal Stress Responses in Drosophila melanogaster
Source: Front Genet. 2020 Jun 23;11:658. doi: 10.3389/fgene.2020.00658 (PMC7324644; doi:10.3389/fgene.2020.00658)

**A**

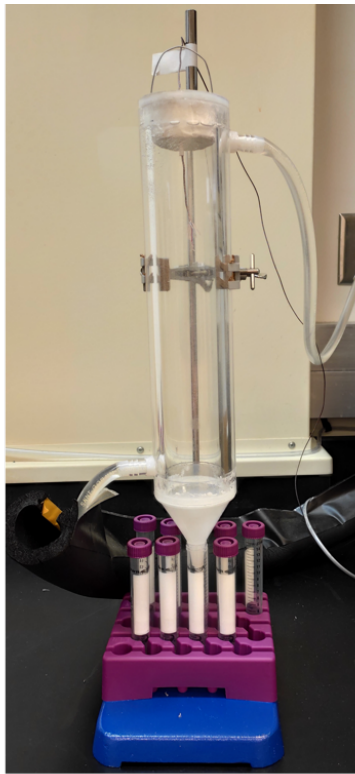

**B**

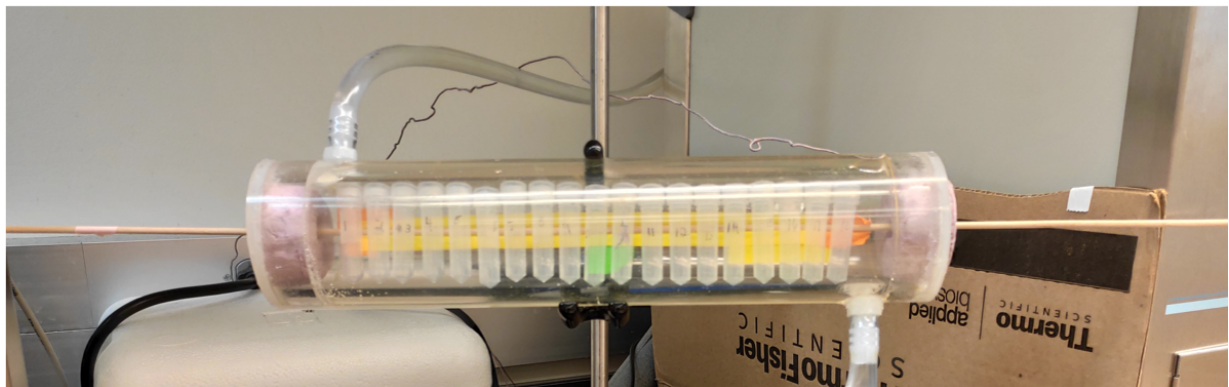

**Supplementary Figure 1. Photograph of apparatus for (A)  $CT_{min}$  and (B)  $CT_{max}$  assays.**

Supplement: Supplementary file 8 [file Image_1.pdf]

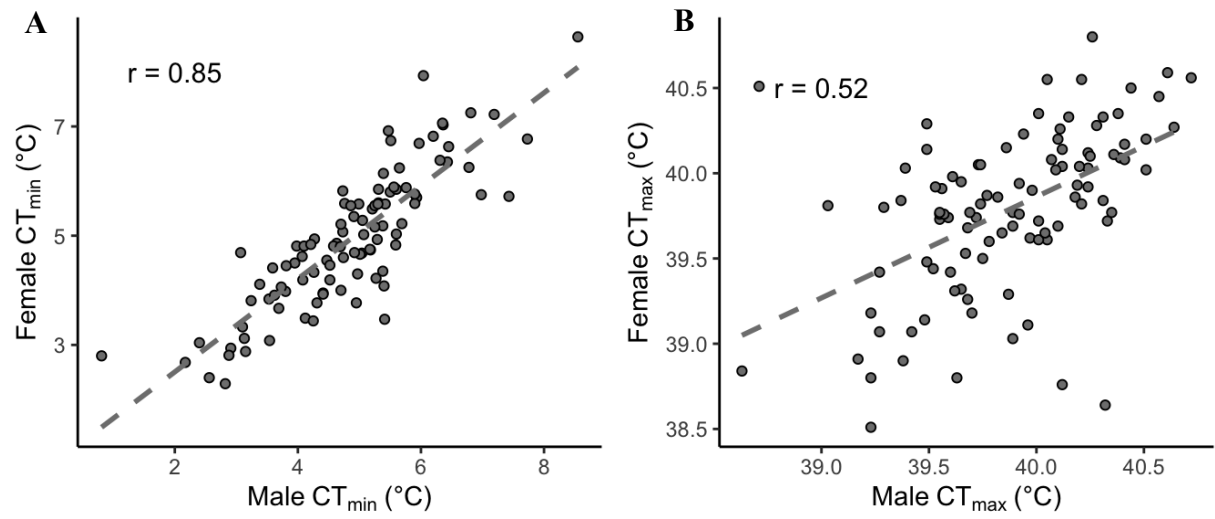

**Supplementary Figure 2. Correlation of thermal limits across sexes. (A)  $CT_{min}$  and (B)  $CT_{max}$ .**

Supplement: Supplementary file 9 [file Image_2.pdf]
