# Supplementary material for: Integrating GWAS and Transcriptomics to Identify the Molecular Underpinnings of Thermal Stress Responses in Drosophila melanogaster
Source: Front Genet. 2020 Jun 23;11:658. doi: 10.3389/fgene.2020.00658 (PMC7324644; doi:10.3389/fgene.2020.00658)
Supplement: Supplementary file 10 [file Image_3.pdf]

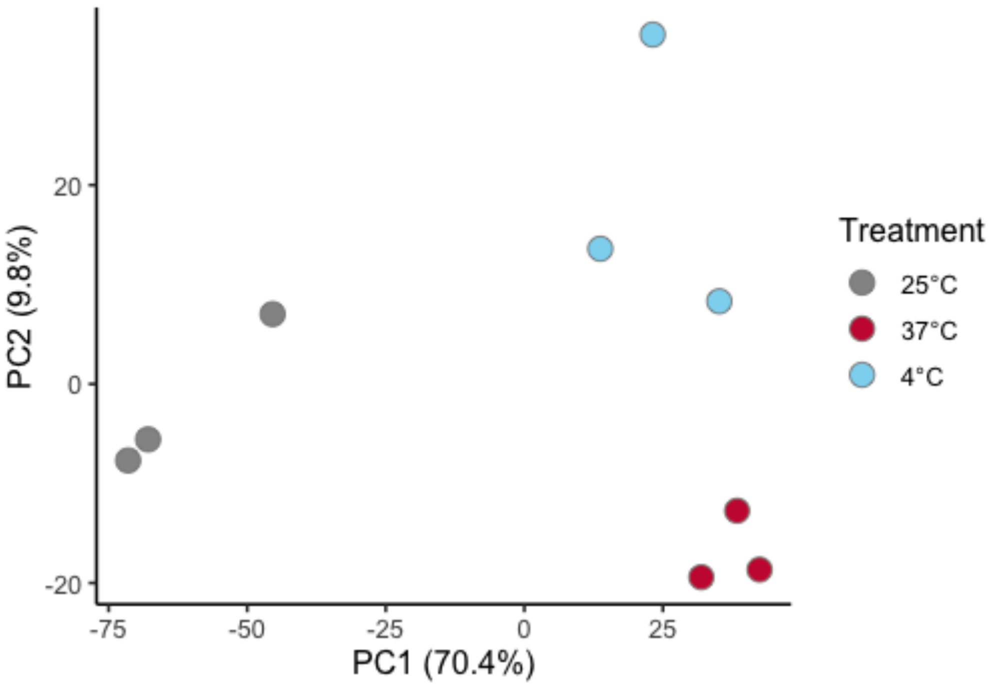

Supplementary Figure 3. Principal component analysis of RNA-seq data. The replicates for each condition clustered together, with the first principal component describing the majority of the variance (70.4%) and separating the control from the temperature stress treatments. The second principal component describes 9.8% of the variance.
